# Establishment of induced pluripotent stem cells derived from patients and healthy siblings of a nevoid basal cell carcinoma syndrome family

**DOI:** 10.1007/s11626-023-00778-y

**Published:** 2023-07-17

**Authors:** Yoji Nakase, Atsuko Hamada, Fumitaka Obayashi, Naoya Kitamura, Tsuyoshi Hata, Tetsuya Yamamoto, Tetsuji Okamoto

**Affiliations:** 1 Department of Oral Oncology, Graduate School of Biomedical and Health Sciences, Hiroshima University, 1-2-3, Kasumi, Minami-Ku, Hiroshima-City, Hiroshima 734-8553 Japan; 2 Department of Oral and Maxillofacial Surgery, Kochi Medical School, Kochi University, Kochi, Japan; 3 Department of Oral Surgery, Kawasaki Medical School, Okayama, Japan; 4 Present affiliation: Kondo Dental Clinic, Medical Corporation Mutsumikai, Okayama, Japan; 5 School of Medical Sciences, University of East Asia, Shimonoseki, Japan

## Abstract

It is known that a nevoid basal cell carcinoma syndrome (NBCCS) is characterized by a combination of developmental abnormalities and a predisposition to form various tumors. Although it is possible to create disease models via gene editing, there are significant potential problems with this approach such as off-target mutations and differences in SNPs. On the other hand, since disease families share common SNPs, research using iPSCs derived from both patients and healthy siblings of the same disease family is very important. Thus, establishment of induced pluripotent stem cells derived from patients and healthy siblings of the same NBCCS family will be of great importance to study the etiology of this disease and to develop therapeutics. In this study, we generated hiPSCs using peripheral blood mononuclear cells derived from the patients and healthy siblings of familial NBCCS with the novel mutation in PTCH1_c.3298_3299insAAG in the feeder- and serum-free culture conditions using SeVdp. In addition, disease-specific hiPSCs such as those expressing the PTCH1_c.3298_3299insAAG mutation could be powerful tools for revealing the genotype-phenotype relationship and pathogenicity of NBCCS.

Nevoid basal cell carcinoma syndrome (NBCCS; OMIM:109,400) is a rare autosomal dominant disorder (Gorlin and Goltz 1960). The symptoms include bifid ribs, palmar pits, and odontogenic keratocysts. Furthermore, patients with NBCCS have a predisposition to various tumors, such as basal cell carcinomas, medulloblastomas, ovariomas, and cardiac fibromas. The estimated rate of NBCCS incidence in Japan is lower than that in other countries; this is one of the reasons that NBCCS is often undiagnosed and partial treatment is administered by a local dermatologist or dentist (Endo *et al*. 2012).

*PTCH1* located on chromosome 9q22 is a gene coding for a hedgehog (Hh) receptor (Hahn *et al*. 1996; Johnson *et al*. 1996). To date, several mutations in *PTCH1* in Japanese patients with NBCCS have been reported, including point mutations, insertions/deletions, and entire deletions (Fujii *et al*. 2003; Nagao *et al*. 2005, 2011; Sasaki *et al*. 2010; Nakase *et al*. 2020). However, no marked hotspots or universal mutations have been observed in *PTCH1* (Lindström *et al*. 2006). Moreover, genotype–phenotype reciprocal relationship has not been reported (Wicking *et al*. 1997).

Disease-specific human induced pluripotent stem cells (hiPSCs) were first reported as iPSCs derived from patient of type II collagenopathy skeletal dysplasia (Okada *et al*. 2015). More recently, a disease model of congenital hepatic fibrosis has been established by mutating hiPSCs with a CRISPR/Cas9 system (Tsunoda *et al*. 2019). Song *et al*. have reported the establishment of a Wilson’s disease (WD)–specific model and retinoids were identified as candidates for supporting the secretion of ceruloplasmin, which is consistently decreased in patients with WD (Song *et al*. 2022). Furthermore, DiGeorge syndrome–specific hiPSCs have been established (Shimizu *et al*. 2022; Song *et al*. 2022). Although it is possible to create disease models via gene editing, there are significant potential problems with this approach such as off-target mutations and differences in SNPs. On the other hand, since disease families share common SNPs, research using iPSCs derived from both patients and healthy siblings of the same disease family is very important. By focusing on the conservation of genetic background and natural cell characteristics, we have reported cases of cleidocranial dysplasia (Hamada *et al*. 2022), Noonan syndrome (Hamada *et al*. 2020b), and Cowden syndrome (Obayashi *et al*. 2022), which have contributed to elucidating the onset and progression of these genetic disorders (Yamasaki *et al*. 2016; Hamada *et al*. 2020a).

In a previous study, we identified a novel mutation in *PTCH1*_c.3298_3299insAAG in familial NBCCS in four generations (Nakase *et al*. 2020). Establishment of induced pluripotent stem cells derived from patients and healthy siblings of the same NBCCS family will be of great importance to study the etiology of this disease and to develop therapeutics. In this study, we generated hiPSCs using peripheral blood mononuclear cells (PBMCs) derived from the patients and healthy siblings of familial NBCCS with the novel mutation in *PTCH1*_c.3298_3299insAAG using the methods described previously (Nakase *et al*. 2020). The Ethics Committee of Human Genome/Gene Analysis Research at Hiroshima University approved this study (approval numbers: hi-58 and hi-72). Informed consents were taken by all participants before the enrollment of this study.

The results of direct sequencing of DNA purified from NBCCS-hiPSCs showed that the *PTCH1*_c.3298_3299insAAG mutation was maintained (Fig. 1). The established NBCCS-hiPSCs were positive for pluripotent markers such as *OCT3/4*, *NANOG*, *REX1*, and *SOX2*, and negative for Sendai virus vector (SeVdp) in the PCR analysis (Fig. 2*A*). The established NBCCS-hiPSCs exhibited differentiation ability into three germ layers in embryoid and teratoma formation assays. In the embryoid assay differentiated NBCCS-hiPSCs expressed b-III tubulin, smooth muscle actin (SMA), and a-fetoprotein (AFP) (Fig. 2*B*). Immunohistochemical analysis revealed that the teratomas differentiated from NBCCS-hiPSCs included gastrointestinal epithelium, which was an endodermal lineage marker, cartilage, which was a mesodermal lineage marker, and neurons, which were ectodermal lineage markers (Fig. 2*C*). During keratinocyte induction, immunocytochemical analysis (ICC) revealed that differentiated cells were positive for TP63 and Nestin, and negative for KRT5 at day 5, and then, differentiated cells became positive for KRT5 in addition to TP63, and negative for Nestin at day 21 (Fig. 3*A*). In western blotting (WB), the expression of KRT5 was gradually increased during keratinocyte induction (Fig. 3*B*). The proliferative potential of keratinocytes derived from NBCCS-hiPSC was significantly higher than that of WT-hiPSC-derived keratinocytes (Fig. 3*C*). In a cartilage differentiation assay, both NBCCS-hiPSCs and WT-hiPSCs had the potential to differentiate into cartilage-like tissue. However, NBCCS-hiPSC-derived cartilage showed lower staining intensity with Alcian blue/PAS (AB) than WT-hiPSC-derived cartilage (Fig. 3*D*). The established NBCCS-hiPSCs were free from *Mycoplasma* infection, which was checked using the e-Myco™ Mycoplasma PCR Detection kit (iNtRON Biotechnology, Gyeonggi-do, Republic of Korea) according to the manufacturer’s protocol (data not shown).iPSCs have been intensively studied and the generation of iPSCs was first described (Takahashi *et al*. 2006). For disease-specific iPSCs, it was reported that in teratomas with a homozygous *PTCH1* mutation, medulloblastoma-like tissue was larger than in teratomas heterozygous for the *PTCH1* mutation, which indicated the importance of PTCH1 in medulloblastoma formation and Hh-related tumors (Ikemoto *et al*. 2020; Nagao *et al*. 2022). Furthermore, the Hh signaling pathway plays an essential role during embryogenesis and maintains stem cell populations in certain adult tissues (Yang *et al*. 2008; Bailey *et al*. 2009). In addition, the Hh pathway also plays an important role in carcinogenesis and is thought to be a therapeutic target in cancer (Michimukai *et al*. 2001; Hooper and Scott 2005). Vismodegib, an inhibitor of smoothened (SMO) that is also a component of the hedgehog signaling pathway, is the first oral therapeutic agent targeting the Hh pathway in medulloblastomas in adults (Von Hoff *et al*. 2009; Robinson *et al*. 2015). Despite its positive outcomes in clinical trials, there are many challenges regarding adverse side effects and acquisition of resistance (Basset-Seguin *et al*. 2015; Robinson *et al*. 2015). Therefore, establishing Hh-related tumor models, including NBCCS-iPSCs, holds promise for the screening of more effective therapies. In addition, disease-specific hiPSCs such as those expressing the *PTCH1*_c.3298_3299insAAG mutation could be powerful tools for revealing the genotype–phenotype relationship and pathogenicity of NBCCS.

**Figure 1.**
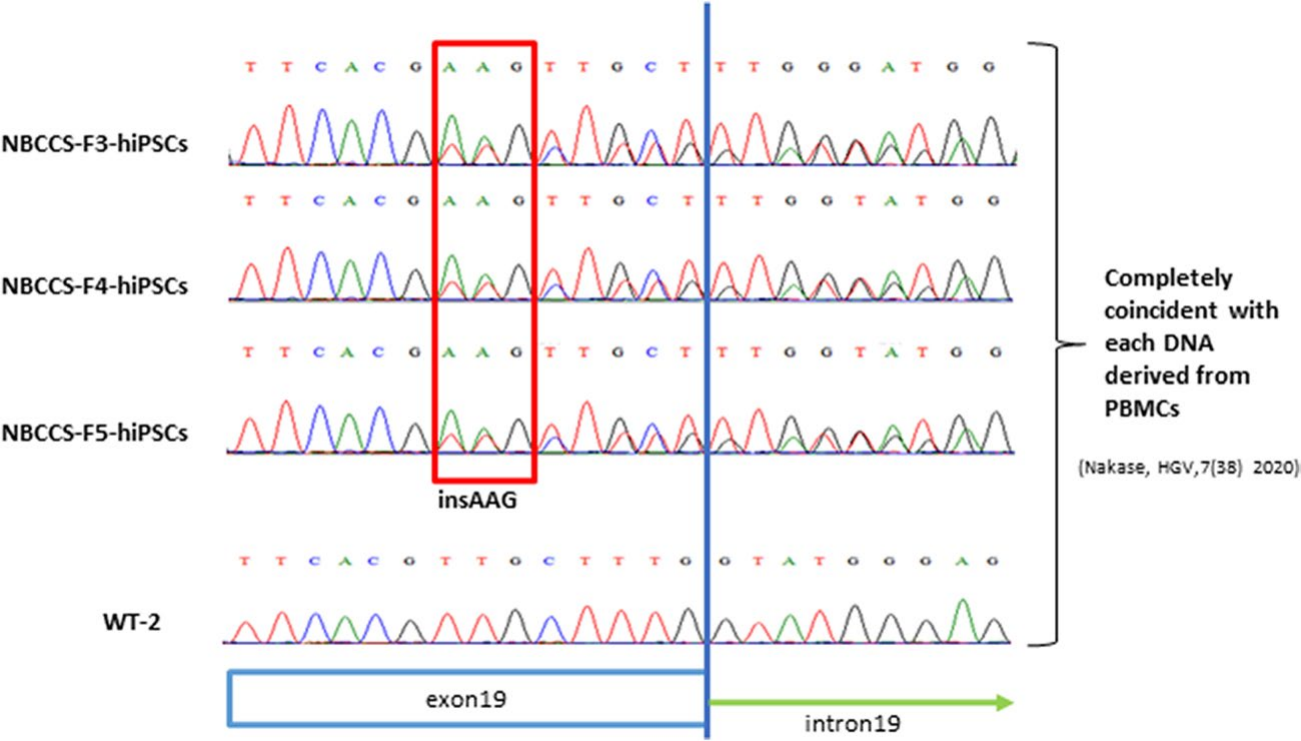
*PTCH1* mutation of familial NBCCS-hiPSCs. The purified polymerase chain reaction (PCR) products of *PTCH1* exon19 were screened by Sanger sequencing using the PowerPlex 16 system (Promega Corporation, Madison, WI), and analyses were performed with the ABI PRISM 3100 Genetic analyzer and Gene Mapper v3.5 (Applied Biosystems). The results of direct sequencing of *PTCH1* exon 19 are shown. Though an insertion was not detected in WT-hiPSC, an AAG insertion was detected between coding sequences 3298 and 3299 in NBCCS-F4-, F5-, and F6-hiPSC.

**Figure 2.**
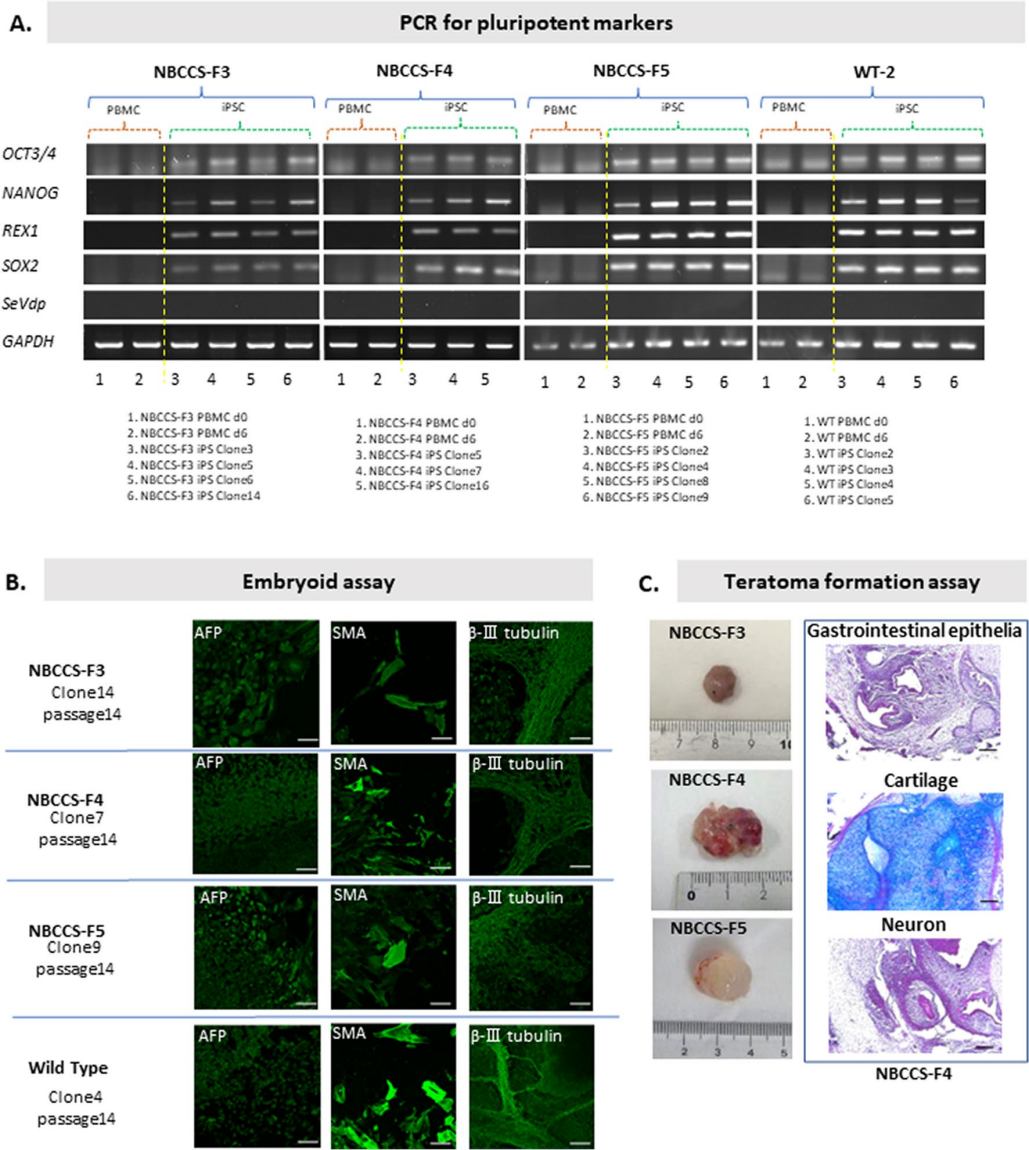
Assay of pluripotency and differentiation ability of NBCCS-hiPSCs. All procedures are performed under feeder-, serum-, and integration-free conditions. Briefly, after separating and culturing PBMCs in serum-free RD6F (Sato *et al*. 1987) supplemented with IL-2 (CELEUK; Takeda Pharm., Osaka, Japan) (Okamoto *et al*. 1996), for 6 d, PBMCs were reprogrammed with SeVdp (KOSM) 302 L (Nishimura *et al*. 2017), which does not integrate into the host genomic background, and further cultured in hESF9 serum-free medium on Laminin-E8 (Nippi, Tokyo, Japan)-coated dish (Hamada *et al*. 2020a). (***A***) PCR for pluripotent makers was performed with TRIzol RNA Isolation Reagents (Thermo Scientific, Waltham, MA), RNA-to-cDNA master mix (Applied Biosystems, Carlsbad, CA), KOD-FX Neo (Toyobo, Osaka, Japan), and ChemiDoc Touch Imaging System (Bio-Rad, Hercules, CA). (***B***) To confirm the differentiation ability of the hiPSCs, embryoid assay and teratoma formation assay were performed. Briefly, for embryoid assay, undifferentiated NBCCS-hiPSCs were cultured in a low-attachment dish, in which gathered sphere formed embryoids. The embryoids were transferred onto a gelatin-coated dish and cultured for 3 wk. After differentiation, immunocytochemistry (ICC) was performed, and fluorescence images were captured using a Zeiss inverted LSM 700 confocal microscope (Carl Zeiss GmbH, Jena, Germany). (***C***) For teratoma formation assay, undifferentiated NBCCS-hiPSCs were injected into the dorsal flank of SCID (CB17/Icr-Prkdcscid/CrlCrlj) mice, and dissected tumors were stained with hematoxylin/eosin (HE) and AB, and histologically analyzed using a Nikon ECLIPSE E800 microscope (Nikon Corporation, Tokyo, Japan) and photographed with a Leica DC500 camera (Leica Microsystems AG, Wetzlar, Germany). Each *bar* indicates 100-µm length.

**Figure 3.**
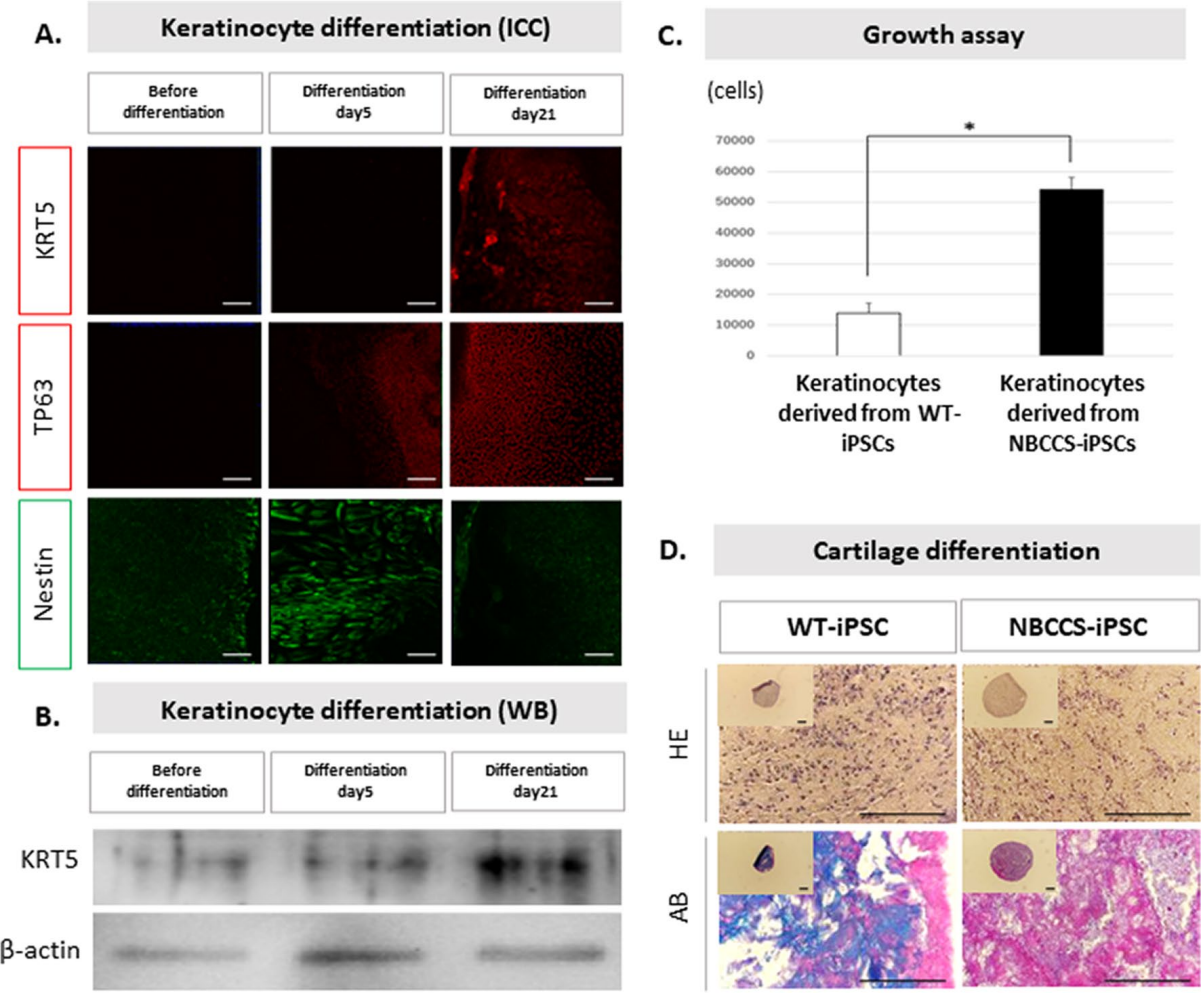
Disease modeling of NBCCS using NBCCS-hiPSCs. (***A*** ~ ***C***) For keratinocyte differentiation (Kogut I, 2014), NBCCS-iPSCs were seeded at 1 × 10^5^ cells/well in a 35-mm dish coated with Laminin-E8, cultured in hESF9 medium with Rock inhibitor (10 μM) (Y-27632, Wako, Osaka, Japan) for 24 h, and then cultured in hESF6 for 2 d. After 24 h of culture in hESF6 with dickkopf (DKK)-1 (10 ng/mL) (R&D, 5439-DK), the culture was switched to hESF6 medium and further cultured for 2 d. After 5 d of culture in hESF6 with both BMP4 (1 ng/mL) (314-BP, R&D, Minneapolis, MN) and all-trans-Retinoic Acid (1 μM) (182–01,111, Wako), the cell was cultured in basal medium which is mixture of MCDB 153HAA (Research Institute for the Functional Peptides, Yamagata, Japan) and RD medium with 6 factors, bovine brain extract (5 μg/mL) (BBE, Coggin Bio, Saitama, Japan), and EGF (12 ng/mL) (R&D, 236-EG) for 16 d. (***A***) To confirm the keratinocyte differentiation, ICC was performed using the following antibodies: TP63 (ab124762, Abcam, Cambridge, England), KRT5 (ab75869, Abcam), and Nestin (Santa Cruz, Dallas, TX), at days 0, 5, and 21. (***B***) To confirm the keratinocyte differentiation, WB was performed. After the protein purification using RIPA buffer (20 mM Tris–HCl, 150 mM NaCl, 1 mM EDTA, 1% TritonX-100, pH 7.4) containing protease inhibitor and phosphatase inhibitor, WB was performed using antibodies, KRT5 and b-actin. (***C***) For growth assay of induced keratinocytes, keratinocytes were seeded in a 24-well plate at the density of 1 × 10^4^ cells/well, and cultured for 5 days, and cells were dispersed with Trypsin/EDTA, and cell numbers were counted on a Coulter counter Z1 (Beckman Coulter, Brea, CA). *Asterisk* indicates *P*-value < 0.03. (***D***) NBCCS-hiPSC- and WT-hiPSC-derived embryo bodies were differentiated into mesenchymal stem cells and then induced into cartilage in the same method, described previously (Hamada *et al*. 2022). Dissected cartilages were stained with HE and AB, and histologically analyzed using a Nikon ECLIPSE E800 microscope. Each *bar* indicates 100-µm length.
